# Different Concentrations of Ropivacaine under Ultrasound Guidance on Quadratus Lumbar Muscle Nerve Block in Elderly Patients with Hip Replacement

**DOI:** 10.1155/2021/9911352

**Published:** 2021-12-22

**Authors:** Yi Han, Xiaoyuan Chen, Panpan Mi, Yunzhao Ji, Xiangan Meng, Pengbo Han, Jingyi Zhang

**Affiliations:** ^1^Department of Anaesthesiology, Hebei PetroChina Central Hospital, Langfang, Hebei Province, China; ^2^Department of Orthopaedics, Hebei PetroChina Central Hospital, Langfang, Hebei Province, China; ^3^Operating Room, Hebei PetroChina Central Hospital, Langfang, Hebei Province, China; ^4^Department of Ultrasound, Hebei PetroChina Central Hospital, Langfang, Hebei Province, China; ^5^Department of Traditional Chinese Medicine, The Second Affiliated Hospital of Xi'an Medical University, Xi'an, Shan'xi Province, China; ^6^Department of Anesthesiology, The Second Affiliated Hospital of Xi'an Medical University, Xi'an, Shan'xi Province, China

## Abstract

**Objective:**

To compare the effect of ropivacaine in different concentrations under ultrasound guidance on lumbar muscle nerve blocking in elderly patients undergoing hip replacement surgery.

**Methods:**

60 elderly patients underwent hip replacement in our hospital over a period of April to December of 2019 were equally randomized into control and observation groups, with 30 each. Patients in the control group and observation group received 0.5% and 0.25% ropivacaine to block psoas muscle nerve, respectively. The anesthetic effect of ropivacaine at different concentrations was evaluated by time of sensory block onset and recovery and time of motor block onset and regression, blood pressure, heart rate, visual analogy scale, and postoperative nerve blocking degree.

**Results:**

The onset time of sensory and motor block in the observation group was dramatically higher than that in the control group (*P* < 0.05), while the recovery time of sensory and motor was significantly shorter than that of the control group (*P* < 0.05). The heart rate in the observation group was notably lower than that in the control group, while the average blood pressure was remarkably higher (*P* < 0.05). After surgery, the degree of nerve block in the observation group was much lower compared with the control group (*P* < 0.05), while no marked difference in the visual analogue scale in the control group before and after surgical intervention was observed (*P* > 0.05).

**Conclusion:**

The 0.25% ropivacaine method has distinctive advantages over 0.50% ropivacaine psoas nerve anesthesia in hip replacement surgery in elderly patients.

## 1. Introduction

Hip replacement remains one of the main options to relieve hip pain in elderly patients [1–3]. Through surgical intervention, the patient's hip bone function impairment caused by fractures or deformities can be improved to a certain extent, thereby significantly improving their quality of life [4–6]. However, due to factors such as age and physical fitness, elderly patients' hip replacement surgery has higher requirements for the type and dosage of anesthetics [7, 8]. Elderly patients are often accompanied by diseases such as diabetes and hypertension, leading to poor cardiopulmonary function, further reducing their resistance to anesthetics, and bringing great challenges to surgery [9, 10]. In addition, the fluctuation of blood pressure during the operation can cause a variety of complications under the action of anesthetics, which seriously affects the patient's quality of life, making the surgical intervention undesirable [11–13]. Studies have shown that the use of nerve block in hip replacement surgery can effectively alleviate the adverse effects of surgery on patients [14]. Based on this, this study intends to use different concentrations of ropivacaine to conduct nerve block in elderly patients with hip replacement surgery under ultrasound guidance and to evaluate the effects of different concentrations of ropivacaine, which is aimed at providing a certain reference for clinical application. We thus innovatively speculated that the use of ultrasound guidance can be used for real-time monitoring with high accuracy and can be characterized by convenient and fast operation and good safety. Due to the real-time monitoring of the whole process, it can effectively reduce and avoid complications.

## 2. Materials and Methods

### 2.1. General Information

Sixty patients who underwent hip replacement surgery in our hospital between April and December 2019 were collected. All patients were divided into control and observation groups, 30 cases each. Randomisation was computer generated centrally by the Oxford Clinical Trials Service Unit, Oxford, UK, and allocations were obtained by telephone from staff who were not involved in other parts of the trial. Randomisation was stratified by centre with minimisation for sex, age, and ASA (American Society of Anesthesiologists) grade. Patients and investigators were not masked to treatment assignment.

### 2.2. Inclusion and Exclusion Criteria

Inclusion criteria are as follows: (1) the ASA classification of patients was I-III; (2) patients who were fully aware of the study; (3) patients who have signed a consent form accompanied by family members; and (4) this study was approved by the ethics committee of our hospital (date of approved by ethic committee: June 2019, Approved No. of ethic committee: 201902/2594).

Exclusion criteria are as follows: (1) with severe inflammation, (2) with neurological diseases, (3) with allergic constitution, (4) with mental disorders, and (5) patients who did not cooperate with clinical follow-up.

### 2.3. Methods

The patient was required to fast for 8 hours prior the operation and was prohibited from taking any drugs. The patient's various indicators were checked before surgery, and their blood pressure and heart rate and other vital signs were monitored in real time. After the patient entered the operating room, he was provided an oxygen mask, and a venous channel was established. The patient's affected side was facing upwards, and ultrasound scanning was used to scan the plane perpendicular to the spine to observe the L3, L4, and L5 transverse processes and the psoas major muscle. Under the assistance of ultrasound, a needle was performed at the gap between the patient's L3 and L4 to suck back the cerebrospinal fluid.

After no abnormal reaction occurred, the control group was injected with a total of 3.00 mL of 0.50% ropivacaine (normal saline : 0.75%ropivacaine = 1 : 2) at a rate of 0.12 mL per second, while the observation group was 3.00 mL of ropivacaine with a concentration of 0.25% (normal saline : 0.75%ropivacaine = 2 : 1), and the needle was withdrawn after the injection. The patient's affected side was kept facing upwards. After 15 minutes of lumbar anesthesia, the hip replacement surgery started if the required anesthesia was reached, and 0.25 mL of ropivacaine should be appropriately supplemented according to the patient's response during the operation. For patients who did not reach the surgical requirements after 15 minutes, 4.00-5.00 mL 0.25% ropivacaine should be added until the surgical requirements were met. Ropivacaine was purchased from Guangdong Jiabo Pharmaceutical Co., Ltd., SFDA approval number H20113381.

### 2.4. Observation Indicators

The onset time and recovery time of sensory block and the time of onset and recovery time of motor block between the two groups were compared; the blood pressure, heart rate, and visual analog scores of the two groups before and after surgery were compared; the degree of nerve block of the two groups after surgery was compared (grade 0: no block; grade I: the patient can lift the leg, bend the knee, and move the ankle joint; grade II: the patient can move the ankle joint but cannot bend the knee; grade III: the patient's lower limbs cannot move).

### 2.5. Statistical Analysis

The statistical analysis was performed by IBM SPSS version 20.0. The mean and standard deviation (SD) was calculated and reported as quantitative variables, and the statistical difference in mean value was tested using paired *t* test and independent *t* test, while *χ*^2^ test was performed to evaluate statistical significance in enumeration variables. A *P* value of <0.05 was considered as statistically significance. The graphs were plotted using Excel.

## 3. Results

### 3.1. Baseline Information

All the patients were comparable with regard to age, gender, and weight (*P* > 0.05, Table 1).

### 3.2. The Effect of Different Concentrations of Ropivacaine on Patients' Sensory and Motor Block

The onset time and recovery time of different concentrations of ropivacaine on elderly patients undergoing hip replacement surgery are shown in Table 2. The onset time of sensory and motor block in the observation group (0.25% ropivacaine) was significantly slower than that in the control group (0.50% ropivacaine) (*P* < 0.05), while the recovery time of sensory block and motor block was significantly faster than that in the control group (*P* < 0.05).

### 3.3. The Effect of Different Concentrations of Ropivacaine on Patients' Heart Rate and Blood Pressure

After anesthesia and surgery, the heart rate of patients in the observation group (0.25% ropivacaine) was significantly lower than that of the control group (0.50% ropivacaine) (*P* < 0.05, Table 3).

After 10 minutes of anesthesia and after surgery, the average blood pressure of patients in the observation group (0.25% ropivacaine) was remarkably higher than that of the control group (0.50% ropivacaine) (*P* < 0.05, Table 3).

### 3.4. The Effect of Different Concentrations of Ropivacaine on the Visual Analog Score of Patients

Before and after anesthesia and after surgery, no marked difference was noticed in the visual analog scale of the observation group (0.25% ropivacaine) and the control group (0.50% ropivacaine) (*P* > 0.05, Table 4).

### 3.5. Comparison of the Degree of Nerve Block of Patients with Different Concentrations of Ropivacaine

After surgery, the degree of nerve block in the observation group (0.25% ropivacaine) was dramatically lower than that in the control group (0.50% ropivacaine) (*P* < 0.05, Table 5).

## 4. Discussion

Hip joint disease is increasingly popular in elderly patients, and these patients are usually more susceptible to the side effects of surgery. Clinical interventions, especially the type and dosage of anesthetics, can severely affect their prognosis [15]. For this reason, optimal anesthesia plan in surgery can effectively reduce the pain caused by clinical intervention and is of great significance to improve the prognosis of patients. In clinical treatment, anticoagulant drugs are aimed at improving blood circulation in the diseased part of the patient. Ropivacaine is an amide drug that can be used to block local nerves in patients during surgery [16]. Because of its rapid onset and low toxicity, ropivacaine has also received increasing attention in clinic research [17].

We found in this study that the onset time of sensory and motor block in the observation group was observably longer than that in the control group, while the recovery time of sensory block and motor block was noticeably shorter than the control group. This suggests that as the concentration increases, the efficiency of ropivacaine in blocking the quadratus lumbar muscle nerve is higher, and the time it takes for the patient to recover from postoperative sensory and motor block is also longer. The visual analog score results showed that there was no marked difference between the visual analog scores of the observation group and the control group before and after the surgical intervention. This indicates that albeit high concentrations of ropivacaine are more effective in anesthesia for patients, there is a marginal value in clinical practical applications, that is, too high concentrations of ropivacaine cannot further enhance its anesthesia effect on patients. On the contrary, it will increase the recovery time required for patients after surgery, thereby increasing the risk of poor prognosis for patients. Moreover, the heart rate of the observation group during the operation was much lower than that of the control group (*P* < 0.05), and the average blood pressure was notably higher than that of the control group. This shows that compared with 0.50% ropivacaine, the use of ropivacaine at a concentration of 0.25% has a smaller impact on the blood flow of the patient and makes the blood flow more stable. Studies have shown that there is a remarkable correlation between the type and dosage of anesthetics and blood pressure fluctuations during surgery. Excessive blood pressure fluctuations can easily cause multiple complications in patients and seriously affect the prognostic quality of life of patients, leading to an undesirable outcome [18]. Therefore, in terms of safety, compared with 0.50% ropivacaine, the use of 0.25% ropivacaine to block the quadratus lumbar muscle nerve has obvious advantages. Our results were in agreement with that of Tamura et al. [19] who compared the effects of 0.25% and 0.50% of ropivacaine in pediatric laparoscopic inguinal hernia repair and found that 0.25% of ropivacaine was more advantageous in analgesia and other aspects.

Due to the small size of samples selected, this study has certain limitations. After all, we confirmed that 0.25% ropivacaine anesthesia during hip replacement surgery in elderly patients has obvious advantages over 0.50% ropivacaine anesthesia, with regard to the hemodynamics, anesthesia recovery time, and postoperative nerve block. To conclude, we believe that the lumbar plexus block with 0.25% ropivacaine has a high application value in the operation of hip replacement in elderly patients.

## Figures and Tables

**Table 1:** General information of patients.

Groups	Age (years)	Male/female	Height (cm)	Body weight (kg)	BMI (kg/m^2^)	ASA grade (I/II/III)
Control group	69.27 ± 7.32	15/15	170.31 ± 11.51	66.93 ± 7.51	23.76 ± 3.32	2/19/9
Observation group	69.09 ± 6.91	16/14	169.96 ± 11.56	69.93 ± 6.51	23.69 ± 3.63	2/18/10
*t*/*Z*	0.10	0.52	0.12	1.65	0.08	-2.42
*P*	0.968	0.865	0.971	0.746	0.966	0.362

Note: BMI: body mass index; ASA: American Society of Anesthesiologists.

**Table 2:** Effects of different concentrations of ropivacaine on patients' sensory and motor block before and after surgical intervention.

Groups	Sensory block onset time (s)	Sensory block recovery time (min)	Onset time of motor block (s)	Motor block recovery time (min)
Control group	18.51 ± 2.03	97.31 ± 5.12	7.51 ± 1.31	109.6 ± 9.13
Observation group	27.09 ± 3.91	81.07 ± 3.35	9.87 ± 1.99	99.31 ± 6.21
*t*	10.67	14.54	5.43	5.10
*P*	0.001	0.002	0.001	0.003

**Table 3:** Effects of different concentrations of ropivacaine on patients' heart rate and blood pressure before and after surgical intervention.

Index	Time	Control group	Observation group	*t*	*P*
Heart rate (times/min)	Before anesthesia	67.56 ± 6.96	67.57 ± 6.95	0.01	0.963
	3 min after anesthesia	74.35 ± 6.22	68.59 ± 5.97	3.66	0.001
	10 min after anesthesia	77.93 ± 6.29	69.03 ± 6.13	5.55	0.002
	30 min after anesthesia	75.91 ± 7.36	70.12 ± 7.12	3.1	0.014
	After surgery	76.42 ± 5.63	68.39 ± 6.63	5.06	0.003
Mean blood pressure (mmHg)	Before anesthesia	96.29 ± 7.93	96.07 ± 6.93	0.11	0.974
	3 min after anesthesia	92.89 ± 6.71	95.19 ± 7.13	1.29	0.846
	10 min after anesthesia	83.69 ± 7.27	91.37 ± 6.97	5.26	0.001
	30 min after anesthesia	83.72 ± 9.35	91.37 ± 6.09	3.76	<0.01
	After surgery	83.81 ± 6.59	92.36 ± 6.11	5.21	0.003

**Table 4:** Effects of different concentrations of ropivacaine on the visual analog scores of patients before and after surgical intervention.

Groups	Before anesthesia	1 h after anesthesia	5 h after anesthesia	24 h after surgery
Control group	5.23 ± 1.19	3.12 ± 0.79	1.07 ± 0.51	0.35 ± 0.09
Observation group	5.25 ± 1.13	3.27 ± 0.97	1.23 ± 0.78	0.39 ± 0.11
*t*	0.07	0.66	0.94	1.54
*P*	0.745	0.763	0.879	0.975

**Table 5:** Comparison of the degree of nerve block in patients with different concentrations of ropivacaine after surgery (*n* (%)).

Group	*n*	0 class	I class	II class	III class
Control group	30	0 (0)	9 (30.00)	12 (40.00)	9 (30.00)
Observation group	30	2 (6.67)	17 (56.67)	10 (33.33)	1 (3.33)
*χ* ^2^		11.04			
*P*		0.001			

## Data Availability

All data are available from the corresponding author on request.
